# Three-dimensional spinal and pelvic posture in professional saxophone players

**DOI:** 10.13075/ijomeh.1896.02934

**Published:** 2026

**Authors:** Ewa Puszczalowska-Lizis, Danuta Smolag

**Affiliations:** 1 University of Rzeszów, Collegium Medicum, Faculty of Health Sciences and Psychology, Rzeszów, Poland; 2 University of Rzeszów, University Center for Research and Development in Health Sciences, Rzeszów, Poland; 3 Silesian Center for Rehabilitation and Prevention, Ustroń, Poland

**Keywords:** occupational exposure, spine, three-dimensional, spinal diseases, musculoskeletal system, DIERS formetric III 4D

## Abstract

**Objectives:**

The psychophysical demands of playing a musical instrument at a professional level pose significant challenges to the musculoskeletal system. Maintaining a position adapted to the instrument for long hours, every day and for many years may result in future postural abnormalities and chronic health problems. This study evaluated the 3-dimensional spinal and pelvic posture of professional saxophone players compared to non-musician controls.

**Material and Methods:**

The study included 60 men aged 20–30 years: 30 professional saxophone players with an average playing experience of mean (M) ± standard deviation (SD) 12.57±2.06 years (the study group) and 30 healthy non-musicians (the control group). Sagittal and frontal spinal posture was assessed using the DIERS formetric III 4D system. Statistical analysis was performed using Student's t-test for independent samples, the Mann-Whitney U test, and Pearson's χ^2^ test.

**Results:**

The professional saxophonists had significantly lower kyphotic angle ICT-ITL (cervicothoracic and thoracolumbar inflection points) max values compared to the control group (p < 0.001), indicating a tendency toward a flatter physiological thoracic curve. Additionally, reduced thoracic kyphosis (hypokyphosis) was more frequently observed in the study group (p = 0.049). No significant differences between the groups were found regarding frontal plane parameters, such as lateral deviation or pelvic obliquity (p > 0.05).

**Conclusions:**

While professional saxophone playing does not alter the direction of spinal deviations, it significantly reduces the thoracic kyphosis angle, predisposing musicians to hypokyphosis. These findings highlight the need for specialised postural training and preventive rehabilitation strategies tailored specifically to the ergonomic demands of saxophone performance.

## Highlights

- DIERS formetric III 4D device detected postural differences in saxophonists vs. controls.
- Saxophonists had lower ICT–ITL max kyphosis angle (p < 0.001).
- Thoracic kyphosis pattern differed significantly between groups (p = 0.049).

## INTRODUCTION

The psychophysical demands of playing a musical instrument at a professional level place significant burdens on the musculoskeletal system. The complex and often repetitive tasks during practice and musical performance increase the risk of musculoskeletal strain [1–4]. Maintaining a position adapted to the instrument for many hours, every day, for many years, may result in postural abnormalities and chronic health problems in the future. An analysis of the available literature indicates that most studies on this topic concern musicians playing string, wind and keyboard instruments, without specifying exact types. It is assumed that job insecurity, irregular performances, the weight and unergonomic shape of instruments, as well as stress and psychosomatic tension associated with performance, may have a significant relationship with the incidence of musculoskeletal disorders and postural defects in this group [5–9].

The lack of scientific research into comprehensive and detailed postural assessment of men with long-term saxophone playing experience prompted the authors to undertake the research and analysis, forming the basis of this work. It is worth emphasizing that the saxophone is held with both hands in front of the body in a relatively symmetrical position. When playing in a standing position, the left upper limb is positioned higher than the right, while the left lower limb is placed slightly forward. This asymmetry may lead to, among other issues, altered tension within the superficial front arm line, the superficial back arm line, and, to a lesser extent, the spiral and lateral lines. Maintaining the instrument in the correct position is facilitated by the thumbs, as well as a neck strap or harness. The saxophone, suspended by a strap or suspenders, should be held alongside the body, parallel to the vertical axis of the trunk. Alternatively, it can be positioned slightly to the side, against the right hip. It is important to hang the saxophone at an appropriate height so that the mouthpiece is level with the lips [10]. The saxophone is an instrument that does not change shape during play. Consequently, saxophonists tend to develop repetitive movement patterns and adopt static body positions that often mimic the shape of their instrument. Therefore, wind instrumentalists represent an occupational group highly vulnerable to postural disorders. These considerations prompted the present study, which aimed to evaluate the 3-dimensional spinal and pelvic posture of professional saxophonists compared to non-musician controls. The research question addressed whether differences exist in spinal posture characteristics, the direction of spinal deviations, and the prevalence of postural abnormalities between professional saxophone players and the non-musician controls.

## MATERIAL AND METHODS

### Participants

The study, conducted in 2024, involved 60 men aged 20–30 years. The study group consisted of 30 saxophone students from the Jazz and Popular Music Departments of randomly selected music academies in Poland (playing experience: mean [M] ± standard deviation [SD] 12.57±2.06 years). The control group comprised 30 age-matched non-musicians students from Jagiellonian University in Kraków, Poland. These institutions were randomly selected, and participants were subsequently recruited from them in consecutive stages of the study.

The following inclusion criteria were established: male sex, age 20–30 years, right-handedness and right-footedness assessed via the *Waterloo Questionnaire – Revised* [11], normal weight status (BMI 18.50–24.99 kg/m^2^) [12], informed, written consent to participate in the study. For the men in the study group, additional inclusion criteria were 10–15 years of saxophone playing experience, playing the saxophone in a standing position, 7 days/week, for ≥5 h/day, and not playing the saxophone within 10 h preceding the study, as well as lack of other musical activities. Exclusion criteria comprised neuromuscular diseases or other chronic illnesses affecting the musculoskeletal system, permanent sequelae of traumatic injuries, other musculoskeletal disorders resulting in balance impairments, congenital skeletal defects (such as asymmetries), and regular participation in competitive sports.

Sample size was estimated using G*Power v. 3.1.9.7 (Heinrich Heine University, Düsseldorf, Germany). For the comparison of continuous variables between 2 independent groups using the Student's t-test or Mann-Whitney U test, assuming a large effect size (d = 0.8), an α level of 0.05, and a desired statistical power of 1 – β = 0.80, the minimum required sample size was calculated to be 26 participants/group. For the Pearson's χ^2^ test, assuming a medium-to-large effect size (w = 0.4, df = 1), α = 0.05, and a power of 0.80, the minimum sample size required was 25 participants/group. Initially, 41 professional saxophone players responded to the recruitment invitation and 41 men were selected as potential controls; however, after applying strict inclusion and exclusion criteria, 30 individuals were ultimately retained in each group. To minimize confounding variables, a matched selection procedure was applied, whereby each participant in the study group was paired with a control subject of similar age and the same sex. Figure 1 illustrates the participant flow through the selection and allocation phases.

**Figure 1.**
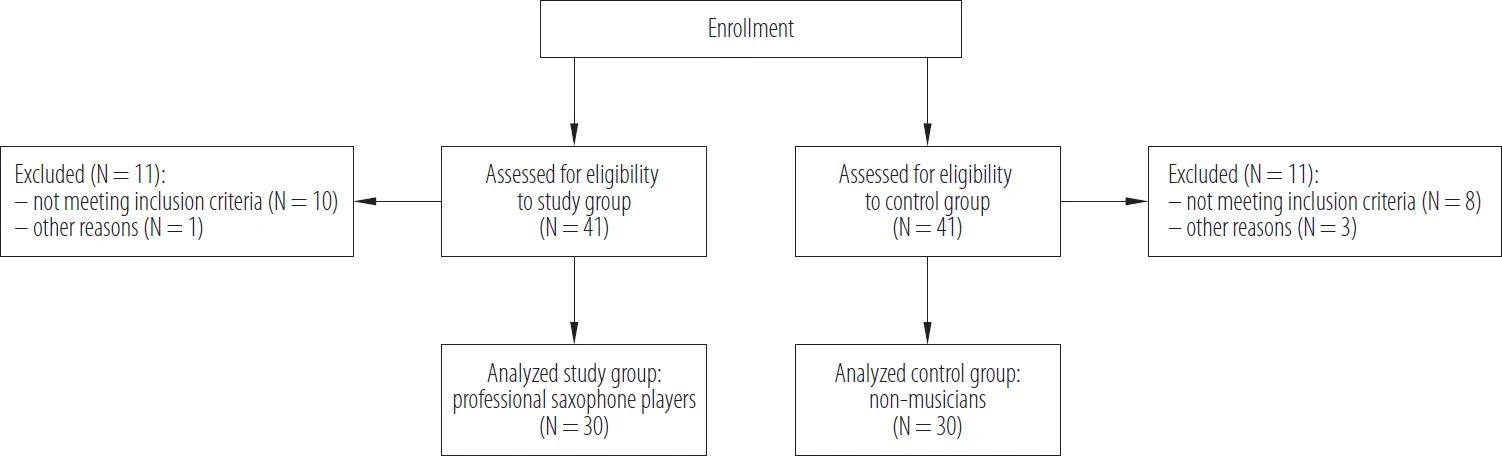
Flow diagram of participant selection and group allocation in the study on musculoskeletal system among professional saxophone players and non-musicians, Poland, 2024

The age of men was M±SD 25.27±2.88 years in the study group, and 24.10±2.50 years in the control group, with no statistically significant differences observed between the groups (p = 0.071). The between-group comparison of somatic characteristics, presented in Table 1, did not reveal statistically significant differences in body mass, height or BMI.

**Table 1.** Comparison of somatic characteristics, absolute values of body posture parameters, and directional deviations in spinal curvature among professional saxophone players (study group) and non-musicians (control group), Poland, 2024

Variable	M±SD		Max–min.		Q_25_		Me		Q_75_		t	Z	p
	study group	control group	study group	control group	study group	control group	study group	control group	study group	control group			
Somatic characteristics													
body mass [kg]	68.37±9.86	71.47±8.01	85.00–46.00	86.00–55.00	60.00	67.00	70.00	72.00	75.00	76.00	–1.34		0.187
body height [cm]	175.00±9.33	176.47±5.88	187.00–150.00	188.00–165.00	168.00	172.00	172.00	175.00	184.00	180.00		0.67	0.501
BMI [kg/m^2^]	22.21±1.95	22.81±1.74	24.98–19.26	24.91–19.03	20.44	21.47	22.12	22.72	24.28	24.28		–1.13	0.258
Body posture parameters													
trunk inclination VP-DM [°]	2.61±3.27	2.49±2.21	16.33–0.02	9.00–0.00	0.26	0.97	1.73	1.67	4.00	3.69		–0.30	0.767
lateral deviation VP-DM [mm]	2.21±1.47	2.23±1.81	4.93–0.02	6.33–0.03	1.22	1.00	1.96	1.54	3.21	3.31		0.35	0.728
pelvic tilt (DL-DR) [mm]	2.82±2.87	3.59±2.41	12.05–0.00	9.96–0.05	0.88	1.85	2.44	3.59	3.67	4.77		–1.63	0.104
surface rotation (RMS) [°]	5.32±3.11	4.03±2.31	11.77–0.89	9.03–0.20	1.67	2.23	5.94	3.90	7.04	5.87		1.60	0.109
pelvic torsion DL-DR [°]	3.78±2.57	2.67±2.09	7.89–0.03	7.24–0.24	1.02	0.59	3.55	2.46	5.85	3.94		1.81	0.070
kyphotic angle ICT-ITL max [°]	42.10±9.23	50.85±7.76	58.19–22.38	66.61–29.08	36.00	46.50	43.75	50.25	48.56	55.00	–3.98		<0.001*
lordotic angle ITL-ILS max [°]	41.47±11.47	44.99±10.05	68.00–21.56	59.35–21.66	35.52	40.35	39.76	46.83	53.36	52.59	–1.26		0.212
Directional deviations in spinal curvature													
trunk inclination VP-DM [°]													
forward	3.18±3.44	2.60±2.24	16.33–0.02	9.00–0.00	1.31	0.98	2.22	1.71	4.00	3.69		305.50	0.579
backward	0.34±0.25	0.90±1.05	0.78–0.04	1.64–0.15	0.25	0.15	0.26	0.90	0.45	1.64		5.00	0.857
lateral deviation VP-DM [mm]													
to the right	1.70±1.85	1.71±1.49	4.56–0.02	5.12–0.03	0.09	0.97	1.36	1.31	3.06	2.32		50.00	0.714
to the left	2.39±1.31	2.69±1.99	4.93–0.54	6.33–0.61	1.50	1.06	2.20	1.64	3.21	4.34		175.50	0.988
pelvic tilt (DL-DR) [mm]													
DR > DL	2.18±2.84	2.50±2.88	9.96–0.05	9.96–0.05	0.40	0.98	1.05	1.78	2.84	2.15		49.50	0.705
DR < DL	3.37±2.87	4.14±2.00	12.05–0.02	9.09–1.03	1.32	2.19	2.85	4.07	4.50	5.36		132.00	0.167
pelvic torsion DL-DR [°]													
DR directed more anteriorly and superiorly than DL	3.78±2.25	2.74±1.99	6.72–0.03	6.02–0.45	1.88	0.59	4.33	2.65	5.60	3.94		52.00	0.312
DL directed more posteriorly and inferiorly than DR	3.78±2.78	2.61±2.23	7.89–0.45	7.24–0.24	0.97	0.54	3.15	2.31	6.88	3.60		113.00	0.140

Q_25_ – lower quartile; Q_75_ – upper quartile; t – value of the Student's t-test for independent samples; RMS – root mean square; Z – value of the Mann-Whitney U test statistics.

DL – left dimple; DM – dimples middle; DR – right dimple; ICT – inflection cervicothoracic point; ILS – inflection lumbosacral point; ITL – inflection thoracolumbar point; VP – *vertebra prominens,* C7.

* p < 0.05.

### Procedures

Spinal posture examinations were performed using the DIERS formetric III 4D device (DIERS International GmbH, Schlangenbad, Germany), which enables optoelectronic recording of the back surface via video-rasterstereography. Degenhardt et al. [13] demonstrated excellent measurement reliability of the device, as intraclass correlation coefficients (ICCs) for most body posture variables ranged 0.75–1.00.

The subjects stood in a relaxed standing position, 3 m from the tripod, with their backs to the projector and video camera. The subject's head was positioned in a neutral position, with their eyes directed horizontally forward. The projector emitted light lines onto the subject's back, and the distortion of the beam was recorded on its surface. The collected data were exported to a computer and processed via dedicated software. The final result was a digital map of the examined body surface, including a 3-dimensional model of the spine. Due to the automatic determination of anatomical landmarks and a correlation model illustrating the relationship between the spine and the surface shape, a spatial representation of the spinal shape and pelvic position was possible. To ensure measurement accuracy and minimize the influence of light reflections, the room in which the tests were conducted was darkened prior to the examinations.

The following variables were selected for analysis:

– trunk inclination VP-DM (°) – deviation of the trunk from the vertical in the sagittal plane – this is the difference in height between VP (*vertebra prominens*, C7) and DM (dimples middle – the midpoint between the left dimple [DL] and the right dimple [DR]) relative to the vertical plane. A positive value of this variable indicates that VP is more anterior than DM, and a negative value indicates that VP is more posterior than DM [14]. The absolute values of this angle should be ≤5° [15];
– lateral deviation VP-DM (mm) – lateral deviation of trunk from vertical plane defined as the lateral deviation of the VP relative to the DM in relation to the vertical plane. Positive values indicate a shift of the VP to the right, while negative values indicate a shift of the VP to the left [14]. The absolute values of this variable should be ≤4 mm [15];
– pelvic tilt DL-DR (mm) – this variable refers to the difference in the heights of the lumbar dimples in the frontal plane. A positive value of this variable indicates that DR is higher than DL, and a negative value indicates that DR is lower than DL [14]. The absolute values of this angle should be ≤4 mm [15];
– surface rotation (root mean square – RMS) (°) – maximum rotation of the spinous processes to the right or left [14]; the absolute values of this variable should be ≤4° [15];
– pelvic torsion DL-DR (°) – this variable is calculated from the mutual torsion of the lumbar dimples (vertical component). With a positive angle difference, DR is directed more anteriorly and superiorly than DL. With a negative angle difference, DL is directed more posteriorly and inferiorly than DR [14]. The absolute values of this variable should be ≤2° [15];
– kyphotic angle ICT-ITL max (°) – the maximum kyphosis angle measured between the tangent to the surface at the inflection cervicothoracic point (ICT, the point of highest surface inclination within the cervical spine above the peak of kyphosis) and the tangent at the inflection thoracolumbar point (ITL, the point of highest negative surface inclination between the peak of kyphosis and the peak of lordosis) [14]. According to Harzmann [15], normal values for this variable range from 45° to 55°;
– lordotic angle ITL-ILS max (°) – this is the maximum angle of lordosis, measured between the tangent to the surface of the ITL and the tangent to the lower inflection lumbosacral point (ILS, the point of the highest positive inclination of the surface between the peak of lordosis and sacral kyphosis) [14]. Normal values of this variable, according to Harzmann [15], range from 32° to 37° for males.

According to the manufacturer's guidelines for the DIERS formetric III 4D device, the prevalence of scoliotic posture and scoliosis was determined based on the values of 3 variables: lateral deviation VP-DM (mm), pelvic tilt DL-DR (mm) and surface rotation (RMS) (°). Scoliotic posture was defined as lateral deviation VP-DM, pelvic tilt <5 mm, and pelvic surface rotation <5°. Scoliosis was defined as lateral deviation VP-DM and pelvic tilt >5 mm, and surface rotation >5° [14,16]. A diagnosis of scoliosis or scoliotic posture required the fulfillment of all 3 clinical criteria; patients who did not meet all 3 benchmarks were classified as unaffected [16].

Body mass and height were measured using the SECA 799 medical scale with a height gauge (SECA GMBH, Hamburg, Germany). Body mass was determined to the nearest 0.1 kg, and body height to the nearest 0.1 cm. Body mass index (weight-to-height index) was calculated from the obtained data.

### Ethics approval

The study protocol received a positive opinion from the Bioethics Committee at the District Medical Chamber in Kraków, Poland (resolution No. 183/KBL/OIL/2023). The study adhered to the principles outlined in the 1964 Declaration of Helsinki and its later amendments. Participants were informed of the purpose, methods, and course of the study, as well as of their right to withdraw consent at any stage without providing a justification. Participation in the study was entirely voluntary, and no financial remuneration was provided. All participants provided written, informed consent for participation in the research.

### Statistics

Data normality was evaluated using the Shapiro-Wilk test. Comparisons of quantitative variable values obtained in the study and control groups were performed using the Student's t-test for independent samples or the Mann-Whitney U test. Dependencies between qualitative variables were examined using Pearson's χ^2^. The threshold of statistical significance was set at p < 0.05. Statistical analysis was performed in Statistica v. 13.3 (TIBCO Software Inc., Palo Alto, California, USA; StatSoft Poland Sp. z o.o., Kraków, Poland).

## RESULTS

As shown in Table 1, a statistically significant difference was observed between the groups regarding the ICT-ITL max kyphosis angle (p < 0.001). The values of this angle in men in the study group were lower than those in the control group. No statistically significant intergroup differences were observed for the remaining variables, including those describing the direction of spinal deviations.

Table 2 shows a statistically significant dependence between the shape of thoracic kyphosis and group membership (p = 0.049), with a higher prevalence of reduced kyphosis observed among men from the study group compared to controls. The remaining variables demonstrated no statistically significant associations.

**Table 2.** Frequency of postural abnormalities among professional saxophone players (study group) and non-musicians (control group), Poland 2024

Variable	Participants (N = 60) [n (%)]		χ^2^	df	p
	study group (N = 30)	control group (N = 30)			
Trunk inclination VP-DM					
anterior	24 (80.0)	28 (93.0)	2.31	1	0.128
posterior	6 (20.0)	2 (7.0)			
excessive	4 (13.0)	4 (13.0)	0.00	1	1.000
norm	26 (87.0)	26 (87.0)			
Lateral deviation VP-DM					
lateral to right	8 (27.0)	14 (47.0)	2.58	1	0.108
lateral to left	22 (73.0)	16 (53.0)			
excessive	4 (13.0)	6 (20.0)	0.48	1	0.488
norm	26 (87.0)	24 (80.0)			
Pelvic tilt DL-DR					
DR > DL	11 (37.0)	10 (33.0)	1.15	2	0.561
DR < DL	18 (60.0)	20 (67.0)			
DR and DL at same height	1 (3.0)	0 (0.0)			
excessive	7 (23.0)	10 (33.0)	0.74	1	0.390
norm	23 (77.0)	20 (67.0)			
Surface rotation (RMS)					
excessive	20 (67.0)	14 (47.0)	2.44	1	0.118
norm	10 (33.0)	16 (53.0)			
Spinal positioning in frontal plane					
scoliosis/scoliotic posture	3 (10.0)	8 (27.0)	5.11	2	0.078
correct	27 (90.0)	22 (73.0)			
Pelvic torsion DL-DR					
DR directed more forwards and upwards than DL	10 (33.0)	14 (47.0)	1.11	1	0.291
DL directed more backward and downwards than DR	20 (67.0)	16 (53.0)			
excessive	19 (63.0)	15 (50.0)	1.09	1	0.297
norm	11 (37.0)	15 (50.0)			
Kyphotic angle ICT-ITL max					
increased kyphosis	2 (7.0)	6 (20.0)	5.14	2	0.049*
norm	16 (53.0)	19 (63.0)			
reduced kyphosis	12 (40.0)	5 (17.0)			
Lordotic angle ITL-ILS max					
increased lordosis	18 (60.0)	23 (77.0)	2.09	2	0.352
norm	8 (27.0)	4 (13.0)			
reduced lordosis	4 (13.0)	3 (10.0)			

Abbreviations as in Table 1.

χ^2^ – Pearson's χ^2^ test statistic.

* p < 0.05.

## DISCUSSION

The present study demonstrates that the ICT-ITL max kyphosis angle is the sole continuous parameter that significantly differentiates professional saxophonists from non-musician controls. Specifically, saxophonists exhibit a smaller thoracic kyphosis angle than the controls. While saxophone playing did not influence the direction of axial spinal deviations, it significantly determined the prevalence of thoracic kyphosis abnormalities. The results of the authors' study, therefore, contradict the earlier findings of Blanco-Pineiro et al. [17], who noted that the saxophone forces musicians to adopt a leaning position, shift the axis of gravity in both the sagittal and frontal planes, and protrude the shoulders forward, thereby deepening the thoracic spine curvature. Shanoff et al. [18] also reported that the most common postural habits among saxophone students at North American universities were a forward head posture and a convex upper back; however, their study was based solely on subjective statements from an online questionnaire.

The authors' findings indicate that a reduction in thoracic kyphosis constitutes the primary feature differentiating the sagittal spinal alignment of professional saxophonists from healthy control peers. This postural flattening appears to be heavily influenced by the prolonged static load of supporting the instrument, which is further exacerbated by the mechanical constraints of performance accessories, specifically standard neck straps (V-necks) and body harnesses. This biomechanical configuration shifts the center of gravity of the saxophonist-instrument system posteriorly relative to the base of support, which subsequently induces elevated tension in both the back muscles and trunk stabilizers. Consequently, a habitual playing posture is established, characterized by the stabilization and co-activation of the trunk and upper limb musculature, thereby facilitating peripheral mobility through proximal stability. This biomechanical compensation exacerbates hyperactivity within the posterior myofascial chain while reciprocally inhibiting the anterior chain, resulting in persistent tonus of the erector spinae muscles. In this context, it is pertinent to evoke Myers' theory of myofascial chains [19], which conceptualizes connective tissue pathways linking disparate anatomical regions into continuous lines of tension.

The reduction of thoracic kyphosis in saxophonists can be elucidated through the interplay of 2 primary myofascial chains: the superficial back line (SBL) and the superficial front line (SFL). The SBL is fundamentally responsible for maintaining an upright posture and counteracting gravitational forward flexion, whereas the SFL governs forward flexion and prevents excessive hyperextension. During saxophone performance, a reduction in myofascial tonus within the ventral thoracic and abdominal regions facilitates the hyperextension and straightening of the thoracic spine, potentially inducing a compensatory increase in tension along the SBL. Consequently, this diminished tension in the anterior trunk stabilizers, coupled with reciprocal hypertonicity of the posterior paraspinal musculature, drives the structural reduction of the thoracic kyphosis curvature. Alterations in sagittal spinal curvatures among musicians playing wind and string instruments have been previously documented, though data specific to saxophonists remain scarce. Studies on other instrumentalists often report flattening of the physiological curves or asymmetrical adaptations due to prolonged, repetitive static loads and instrument weight distribution [5]. The consequences of a reduced thoracic kyphosis (hypokyphosis or a “flat back” tendency in the thoracic region) are clinically significant. Functionally, the physiological curves of the spine act as a natural spring mechanism. A reduction in thoracic kyphosis diminishes the spine's shock-absorbing capacity, thereby increasing axial loading on the intervertebral discs and vertebral bodies [20]. Over time, this structural alteration can accelerate degenerative disc disease and lead to compensatory hypermobility or pain in the adjacent cervical and lumbar segments [21]. Furthermore, a flattened thoracic spine alters the length-tension relationship of the scapulothoracic and erector spinae muscles, potentially precipitating chronic myofascial pain syndrome and muscular imbalances common among professional instrumentalists [9]. It is important to note that the reduced thoracic kyphosis observed in our cohort of professional saxophonists should be characterized as a functional occupational adaptation rather than an immediate structural pathology. While not inherently pathological at this stage, this altered alignment modifies the physiological distribution of axial loads. Consequently, it represents a postural risk factor that could potentially worsen with cumulative years of playing and lead to symptomatic musculoskeletal disorders. Future prospective studies focusing on longitudinal changes in cohorts with extensive professional experience are warranted to track the progression of this adaptation.

As noted above, due to the biomechanical demands of the instrument, the tissues of the SBL may exhibit a predisposition toward adaptive shortening. Given that wind instrument performance necessitates precise ventilatory control, this fascial shortening compels the saxophonist to redirect respiratory expansion predominantly into the anterior chest wall, thereby inducing a compensatory forward protrusion of the ventral torso. Saxophone performance necessitates optimal pulmonary ventilation, precise airflow regulation, and the capacity to generate substantial intraoral pressure [22]. The forceful expulsion of air through the constrained tip opening between the mouthpiece and the reed encounters high aerodynamic resistance. Consequently, this mechanism demands extensive recruitment of the accessory expiratory muscles alongside intensive, coordinated diaphragmatic activity to regulate subglottic pressure. The simultaneous sensorimotor and physiological demands of supporting the instrument, regulating airflow through the mouthpiece, and managing breath support can significantly alter thoracic spine mechanics. Consequently, this multi-tasking leads to hypertonicity within the musculature of the anterior thorax, shoulder girdle, and cervical region [10, 22–24]. According to Norris [25], to ensure full control of the instrument and ease of both inhalation and exhalation, saxophone players retract their heads and avoid excessive thoracic flexion; this leads to changes in the load on the joints of the cervicothoracic junction. Notably, the diaphragm's action significantly affects body posture by reducing the angle of thoracic kyphosis, particularly in individuals engaged in activities requiring intense breath control, such as saxophone playing. The diaphragm is the primary respiratory muscle that increases thoracic cavity volume during contraction to enable inhalation. Its action influences the musculoskeletal system by activating postural muscles, which may reduce thoracic kyphosis. Notably, the diaphragm is closely linked to the deep stabilization system of the spine, including the erector spinae and abdominal muscles. Consequently, the intense respiratory demands of saxophone playing require the diaphragm to act simultaneously as a respiratory muscle and a trunk stabilizer [24,26]. Furthermore, deep inhalation – particularly in saxophonists – triggers enhanced activation of both respiratory and stabilizing muscles. This elevates tension in the erector spinae, potentially reducing thoracic kyphosis [22,24].

Diaphragm activity during saxophone playing requires deep, controlled inhalation. This process expands the thoracic cavity and stretches the anterior trunk musculature, such as the intercostal and pectoral muscles, thereby promoting thoracic spine extension. Furthermore, the expansion of the rib cage during intense inspiration increases intrathoracic volume, potentially reducing thoracic kyphosis [27]. Kocjan et al. [27] demonstrated that inspiratory diaphragmatic activity increases intra-abdominal pressure, acting as a natural corset to stabilize the spine, particularly the lower thoracic and lumbar segments. This stabilization functions synergistically with the rectus abdominis. Beyond its respiratory function, the diaphragm contributes significantly to postural balance through synergistic co-activation with other deep core musculature, including the iliopsoas, pelvic floor, and abdominal muscles [27]. This collective stabilizing mechanism provides enhanced structural support to the spine, potentially reducing thoracic kyphosis. Consequently, the intensive diaphragmatic engagement required of saxophonists necessitates a more upright posture to facilitate optimal breath control during performance. Obayashi et al. [28], in a study on swimmers, investigated the interplay between diaphragmatic function, body posture (including the thoracic kyphosis angle), and the activation of posterior musculature, such as the erector spinae. They demonstrated a clear link between these structures, spinal stabilization, and trunk flexion. The authors noted that respiration engages both the back and abdominal muscles, which serve as critical components in postural control. These findings suggest that respiratory muscle training can reduce thoracic kyphosis, likely via enhanced abdominal muscle recruitment. Kapandji [29] demonstrated the complex relationship between the diaphragm and spinal curvatures, highlighting the critical role of the accessory respiratory muscles (the “fourth group”) during forced exhalation. This group comprises the *iliocostalis thoracis*, *longissimus thoracis*, *serratus posterior inferior*, and *quadratus lumborum*. Together with the diaphragm, these muscles constitute a coordinated synergistic-antagonistic system. Because exhalation during saxophone playing is an active process, intense tension in both the primary and accessory expiratory muscles engages the abdominal press. This mechanism generates a rigid, pressurized cylindrical “pillar” that stabilizes the body when supporting a load, in this case, the instrument. Investigating functional disorders of the diaphragm and their impact on the musculoskeletal system, Kolar et al. [30] emphasized that effective respiratory function relies on the entire motor complex, including the shoulder girdle, thorax, abdominal structures, and spine.

In summary, this work can provide a foundation for filling the existing research gap. The presented results may have significant implications for both science and physiotherapy practice, supporting the development of postural prevention programs for saxophone players. It is important to emphasize the need to develop preventive measures aimed at educating those beginning to learn to play an instrument, particularly in the early stages of training, when basic motor patterns are being developed. This will help avoid deficiencies that, if repeated during further musical education, can become consolidated and lead to chronic playing-related musculoskeletal disorders. Consequently, instrumental pedagogy literature should integrate information on proper body posture and musculoskeletal deformity prevention. Ultimately, these findings serve as a valuable springboard for future research integrating physical strain with stress, fatigue, and psychological aspects to provide a holistic framework for professional saxophonists' health.

## CONCLUSIONS

The ICT-ITL max kyphosis angle is the sole continuous parameter that significantly differentiates professional saxophone players from non-musician controls. Specifically, saxophonists exhibit a smaller thoracic kyphosis angle than the controls. While saxophone playing did not influence the direction of axial spinal deviations, it significantly determined the prevalence of thoracic kyphosis abnormalities, specifically manifesting as a flattening of the curve (hypokyphosis).
